# A Novel *In Vitro* Model for Microvasculature Reveals Regulation of Circumferential ECM Organization by Curvature

**DOI:** 10.1371/journal.pone.0081061

**Published:** 2013-11-21

**Authors:** Sebastian F. Barreto-Ortiz, Shuming Zhang, Matthew Davenport, Jamie Fradkin, Brian Ginn, Hai-Quan Mao, Sharon Gerecht

**Affiliations:** 1 Department of Chemical and Biomolecular Engineering, Johns Hopkins Physical Sciences-Oncology Center and Institute for NanoBioTechnology, Johns Hopkins University, Baltimore, Maryland, United States of America; 2 Department of Materials Science and Engineering, Johns Hopkins University, Baltimore, Maryland, United States of America; 3 Translational Tissue Engineering Center, Johns Hopkins School of Medicine, Baltimore, Maryland, United States of America; 4 Department of Biomedical Engineering, Johns Hopkins University, Baltimore, Maryland, United States of America; University of California, San Diego, United States of America

## Abstract

In microvascular vessels, endothelial cells are aligned longitudinally whereas several components of the extracellular matrix (ECM) are organized circumferentially. While current three-dimensional (3D) *in vitro* models for microvasculature have allowed the study of ECM-regulated tubulogenesis, they have limited control over topographical cues presented by the ECM and impart a barrier for the high-resolution and dynamic study of multicellular and extracellular organization. Here we exploit a 3D fibrin microfiber scaffold to develop a novel *in vitro* model of the microvasculature that recapitulates endothelial alignment and ECM deposition in a setting that also allows the sequential co-culture of mural cells. We show that the microfibers' nanotopography induces longitudinal adhesion and alignment of endothelial colony-forming cells (ECFCs), and that these deposit circumferentially organized ECM. We found that ECM wrapping on the microfibers is independent of ECFCs' actin and microtubule organization, but it is dependent on the curvature of the microfiber. Microfibers with smaller diameters (100–400 µm) guided circumferential ECM deposition, whereas microfibers with larger diameters (450 µm) failed to support wrapping ECM. Finally, we demonstrate that vascular smooth muscle cells attached on ECFC-seeded microfibers, depositing collagen I and elastin. Collectively, we establish a novel *in vitro* model for the sequential control and study of microvasculature development and reveal the unprecedented role of the endothelium in organized ECM deposition regulated by the microfiber curvature.

## Introduction

The development of functional microvascular vessels requires complex interactions between endothelial cells (ECs), perivascular cells, and the extracellular matrix (ECM) [1], [2]. The ECM is comprised of an abundance of nanometer sized macromolecular ECM proteins. As such, many of the physical interactions between vascular cells and macromolecular components of the ECM occur at the sub-micron scale. EC adhesion to the ECM initiates the angiogenic cascade [3]. Cells detect and respond to the nanoarchitecture of their microenvironment by cytoskeletal reorganization and activated signaling cascades to regulate fundamental cell behaviors. Indeed, it has been shown that surfaces with nano-scale line-grating features affect EC adhesion, alignment, and elongation [4]–[7].

ECs, sitting on their basal lamina, comprise the innermost lining of the vessels, followed by a layer of subendothelial connective tissue and an internal elastic lamina composed of different ECM proteins. These layers together make the tunica intima of blood vessels. Mural cells, along with their ECM, make up the tunica media or middle layer and provide the contractility necessary for vasoreactivity. On top of this layer lays the tunica adventitia, though it is only present in larger blood vessels. [2], [8]–[15]. The specific composition as well as the organization and arrangement of both cellular and ECM components in each layer are necessary for proper microvasculature development, maturation, stability, and function [1], [2].

It has been shown that in microvasculature EC nuclei and cytoskeleton are aligned in the direction of blood flow. However, studies on microvasculature have not analyzed the organization of different ECM components in detail [15]–[17]. By large, the literature that has studied the particular organization of the ECM in native blood vessels is focused on analyzing the elastin and collagen structures in the aorta and other large arteries [10]–[14], [18]. These studies have shown that the structural organization of collagen varies not only between the three layers of the vasculature, but also varies with vessel size and specific location in the body [12].

Overall, the studies agree that each tunica possesses at least two different families of collagen fibrils, with distinctly different organizations. The general arrangement has been found to be close to axial in the adventitia, outer layers having a more pronounced axial orientation transitioning to a circumferential alignment in inner layers [14]. In contrast, the medial layer has been shown to have a nearly perfect circumferential order [11]–[14]. Studies also agree both the internal and external elastic lamina are fenestrated, though both axial and circumferential organization of these layers has been reported [15], [19]. On the other hand, the subendothelium has been found to have a more varied composition. It has been described as a multilayered fabric of collagen, containing distinct layers of both longitudinally and circumferentially aligned fibers [10], [13], [14], [20]; some studies suggesting a layer of longitudinally aligned ECM directly under the media layer, followed by a helically arranged region of connective tissue beneath, and a thin circumferentially aligned layer next to the lumen [14]. However, it is important to note that these findings were all made on large arteries with diameters in the millimeter range.

To date, only a few studies have attempted to model or recreate microvasculature in a full 3D setting *in vitro* [21]–[23], as the majority of models have either focused on the development of capillary beds in natural or synthetic hydrogels [24]–[35], decellularized matrices [36], [37], and electrospun polymer scaffolds [38], [39], or have instead aimed to create vascular grafts typically >3 mm in diameter [40]–[45]. While these models enabled us to study the ECM-driven molecular mechanisms that regulate EC tubulogenesis, they typically support spontaneous and random tubulogenesis (size, shape, organization, etc.). Recent works employ micro-patterning and demonstrate an organized vascular network structure within hydrogels, some of which are able to recruit vascular smooth muscle cells (vSMCs) [46]–[48]. Nonetheless, these systems have limited control over topographical cues presented by the ECM and impart a barrier for the high-resolution, dynamic, and detailed study of vascular organization and specific cell-ECM and multi-cellular interactions.

Tubular polymeric scaffolds have the potential to provide a better and more sophisticated platform to study the microvasculature, but currently are obtainable with diameters in the millimeter range [49]–[51] and are utilized to study graft's mechanical strength [52]–[54]. To recapitulate the microvasculature *in vitro*, the tubular scaffolds must exhibit a physiologically-relevant diameter and sub-micron topography with sufficient mechanical properties, be biocompatible, mediate specific cell adhesion, allow tubular vessel formation, and support multi-cellular interactions.

Endothelial colony forming cells (ECFCs), a subpopulation of endothelial progenitor cells, are known for their proliferative capacity and contribution to functional vessels [55]–[57]. Recently, we revealed that ECFCs deposit ECM proteins, namely collagen IV, fibronectin and laminin, and also assemble it into web-like structures when cultured on Petri dishes [58]. This finding suggests an important role for ECM production by ECFCs in the process of vascular assembly that has not yet been identified.

Here we exploit a three-dimensional (3D) fibrin microfiber scaffold to develop a novel *in vitro* model of the microvasculature that recapitulates ECFC alignment and ECM deposition in a setting that also allows the sequential co-culture of vSMCs. Using this model, we determine that the microfiber curvature affects the circumferential deposition of ECM from ECFCs independently of cellular organization, demonstrating unique opportunities to study microvasculature development and regeneration.

## Materials and Methods

### Cell Culture

Human ECFCs (Lonza, Walkersville, MD) were used for experiments between passages 5 and 9. ECFCs were expanded in flasks coated with type I collagen (BD Biosciences, Franklin Lakes, NJ) in Endothelial Basal Medium-2 (EBM-2; Lonza) supplemented with EGM-2 Bulletkit (Lonza) and 10% fetal bovine serum (FBS; Hyclone, Logan, UT). ECFCs were fed every other day, passaged every 5 to 7 days with 0.05% trypsin/0.1% ethylenediaminetetraacetic acid (EDTA; Invitrogen, Carlsbad, CA). Human vSMCs (ATCC, Manassas, VA) were used between passages 4 and 9 and cultured in F-12K medium (ATCC) supplemented with 0.01 mg/ml insulin (Akron Biotech, Boca Raton, FL), 10% FBS (Hyclone), 0.05 mg/ml ascorbic acid, 0.01 mg/ml transferrin, 10 ng/ml sodium selenite, 0.03 mg/ml endothelial cell growth supplement, 10 mM HEPES, and 10 mM TES (all from Sigma-Aldrich, St. Louis, MO).

### Preparation of 3D fibrin hydrogel microfiber

**Figure 1:**
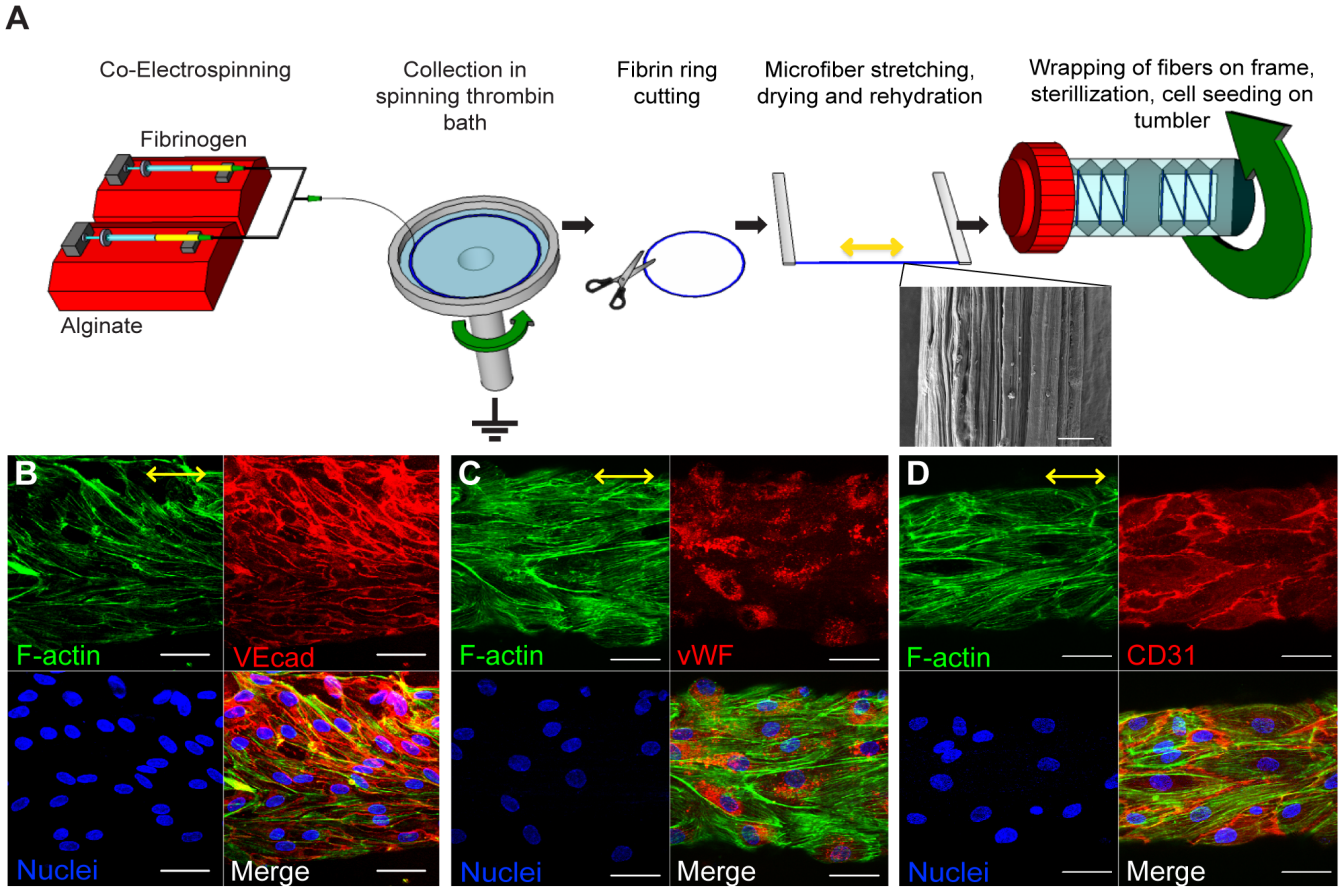
ECFCs attached and aligned on fibrin hydrogel microfibers. (**A**) Schematic of experimental procedure including electrospinning, ECFC seeding, and tumbling. Drawing not to scale. SEM of critical-point dried fibrin microfiber showing aligned topography on the microfiber surface. Scale bar is 10 µm. (**B–D**) Confocal z-stack image reconstructions of ECFCs seeded on fibrin hydrogel microfibers horizontally aligned after 5 days in culture. F-actin (phalloidin staining) is shown in green, EC-specific markers (VECad, CD31, or vWF) in red, and nuclei in blue. Yellow arrows indicate the direction of stretching and nanotopography on microfiber surface. Scale bars are 50 µm. n≥2 per stain with quadruplicates.

Fibrin hydrogel microfibers were generated by a new electrostretching method (**Fig. 1A**) [59]. An aqueous solution of 1.5 wt% alginate (Sigma-Aldrich) was in-line mixed with 2 wt% fibrinogen (Sigma-Aldrich) at feeding rates of 2 ml/h and 1 ml/h, respectively. The mixed solution was then charged with 4 kV electric potential and extruded through a 25-gauge needle. The fibrinogen-alginate solution jet was collected at a distance of 3–5 cm from the needle tip, in a grounded, rotating bath (20 cm diameter, 20–40 rotation/min) containing 50 mM CaCl_2_ solution with 5 units/ml thrombin (Sigma-Aldrich). To generate microfibers with different diameters, collection times were varied from 7–75 min. After spinning, the resulting ring of nanofibers was left in the collection solution for 15 min before being cut once to obtain nanofiber strands. The crosslinked fibrin-alginate nanofibers were then soaked in 0.2 M sodium citrate overnight to remove calcium ions and dissolve alginate. Nanofibers were soaked in water for 30 min to remove sodium citrate, collected as an aligned bundle, stretched to 150% of their initial length, and air-dried for 30 min. Resulting microfibers (**Fig. 1A**) were wrapped around a custom-made plastic frame, then sterilized by soaking in 75% ethanol for 2 min followed by rinsing twice with sterile water. Microfiber diameter was measured from confocal z-stack projections.

### Cell seeding and culture on fibrin hydrogel microfibers

ECFCs were seeded on microfibers of 15 cm length total at a density of 4×10^5^ cells/ml in 5 ml of ECFC media supplemented with 50 ng/mL of VEGF (Pierce, Rockford, IL, USA). The seeding tube was left on a tumbler (Labquake, Dubuque, IA) for 24 h at 37°C to facilitate cell attachment. Frames with ECFC seeded microfibers were then transferred to 35 mm Petri dishes using tweezers, and cultured in the same media in a CO_2_ incubator at 37°C. Media was refreshed every other day thereafter. vSMCs were seeded on 5–7 day ECFC-seeded fibrin microfibers at 1–4×10^5^ cells/ml in 5 ml of 0.5% serum or regular ECFC media, tumbled for 24 hours, and then transferred to 35 mm Petri dishes to continue culture. Media was changed every other day thereafter.

### Preparation of 2D fibrin nanofiber sheets

Fibrin-alginate hydrogel nanofibers were prepared according to the same electrostretching protocol as described above. The collected fibrin-alginate hydrogel nanofibers were wrapped around a modified plastic frame to form a sheet of nanofibers while slightly stretching the nanofibers to ensure proper alignment. The fibrin sheets were placed in a 0.2-M sodium citrate solution overnight to remove alginate, followed by a 30 min wash in water to remove excess sodium citrate. Fibrin sheets were then sterilized with 75% ethanol and rinsed twice with sterile water.

### Cell seeding and culture on 2D fibrin sheets

Cells were seeded by placing 5×10^5^ ECFCs in a concentrated solution of cells (about 2×10^6^ cells/ml) directly on top of the fibrin sheet. After 5 min the cell solution that had filtered through the sheets was collected and reseeded on top of the fibrin sheets. This process was repeated 3 times, after which the same culture media as used for 3D fibers was added to the samples before being placed in a humidified incubator at 37°C in a 5% CO_2_ atmosphere. Media was refreshed every other day up to 5 days of culture.

### Preparation of 3D polyethersulfone (PES) fibers

Solid polymer fibers were prepared as a control according to a modified electrostretching protocol. In brief, PES (Goodfellow Cambridge Limited, UK, Mw 55,000) was dissolved in 30 wt% DMSO and electrospun under an electric potential of 5 kV. The feed rate of PES solution was 12 ml/h to initiate a polymer jet, which was collected in a grounded, rotating ethanol bath (20–40 rotations/min) to extract the solvent. The collection distance was set to 5 cm. After 10 min in ethanol, PES strings were removed from the bath and air dried. After electrospinning, PES fibers were wrapped around a seeding frame similarly to the fibrin hydrogel microfibers. Samples were then plasma-treated for 5 min before soaking for 5 min in a 10 units/ml thrombin in 15 mM CaCl_2_ solution. Thrombin-coated PES fibers were then immersed in a 0.2% fibrinogen solution diluted in 0.9% NaCl for fibrinogen polymerization into fibrin. Excess fibrin coating on the frame and outside of the fibers was removed before sterilization with 75% ethanol for 1–2 min. Samples were rinsed twice with sterile water, after which cell seeding was performed similarly to the fibrin microfibers as described above.

### Actin and microtubule disruption studies

Cytochalasin D or nocodazole were dissolved in DMSO (**Table S1**). ECFCs were cultured on Petri dishes or fibrin microfibers in the same media described above supplemented with either 1 µg/mL cytochalasin D or 3.3 µM nocodazole from either day 0 or day 1 after seeding. F-actin or α-tubulin organization and ECM deposition was analyzed after 1, 2, or 3 days of treatment. Final concentration of DMSO in cell culture medium was kept at 0.1% (v/v). Controls were treated with DMSO alone at the same concentration.

### Immunofluorescence staining and confocal microscopy imaging

Cell-microfiber constructs were fixed with 3.7% formaldehyde (Fisher Chemical, Fairlawn, NJ) for 15 min, permeabilized with 0.1% Triton X-100 solution (Sigma-Aldrich) in 3.7% formaldehyde for 10 min, washed three times with PBS, and incubated for 1 h at room temperature with the indicated primary antibodies (**Table S1**). After rinsing with PBS three times, samples were then incubated with the appropriate secondary antibodies or conjugated phalloidin (**Table S1**) at room temperature for 1 h. Samples were then rinsed with PBS three times, and counterstained with DAPI for 10 min. Z-stack and cross-sectional images were obtained and processed using confocal microscopy (LSM 510 Meta, Carl Zeiss Inc., Thornwood, NY). Epifluorescence images were obtained using an Olympus BX60 microscope.

### Transmission electron microscopy

Samples were prepared for transmission electron microscopy (TEM) analysis as described previously [60]. Briefly, samples were fixed with 3.7% formaldehyde, 1.5% glutaraldehyde in 0.1 M sodium cacodylate, 5 mM CaCl_2_, and 2.5% sucrose at room temperature for 1 h and washed 3 times in 0.1 M cacodylate/2.5% sucrose (pH 7.4) for 15 min each. The cells were post-fixed with Palade's OsO_4_ on ice for 1 h, *en bloc* stained with Kellenberger uranyl acetate overnight, dehydrated through a graded series of ethanol, and then embedded in EPON. Sections of 80-nm were cut, mounted onto copper grids, post-stained in 2% uranyl acetate and Reynolds lead citrate, and viewed using a Phillips EM 420 transmission electron microscope (FEI). Images were captured with an Olympic Soft Imaging Systems Megaview III CCD digital camera.

### Scanning electron microscopy

Hydrogel microfiber samples were first serially dehydrated in 50%, 60%, 70%, 80%, 90%, 95% and 100% ethanol for 15 min in each solution, critical point dried, and then sputter-coated with 8-nm thick Au/Pd. Samples were imaged on a field-emission SEM (JEOL 6700F, Tokyo, Japan).

### Image and statistical analyses

All image analyses were performed on at least 60 measurements per condition. Cytoskeletal alignment angle was calculated by fitting an ellipse to each cell using the LSM 510 software and measuring the angle between the long axis of each ellipse and the longitudinal axis of the microfiber, found by drawing a line at the edges of the microfiber in its image projection. ECM angle of orientation was measured by drawing a line following the ECM deposition and finding the angle between the line and the longitudinal axis of the microfiber. Graphs were plotted with 5–95% confidence intervals. Unpaired two-tailed Welch-corrected *t*-tests were performed where appropriate (GraphPad Prism 5.01, GraphPad Software, San Diego, CA). Significance levels were determined between samples examined and were set at *p<0.05, **p<0.01, and ***p<0.001.

## Results

### ECFC attachment and alignment on fibrin microfibers

We have recently developed a new approach to create aligned hydrogel microfibers using an electrostretching process from various polymer materials [59]. Unique characteristics of the electrostretched hydrogel microfibers are the internal and topographical alignment of the fibrous structure, generated as a result of both electrical field and mechanical shear-induced polymer chain alignment [59]. Furthermore, the microfibers' diameter is controllable and uniform as a result of the bundling and processing of the individual nanofibers composing the microfibers. As fibrin gels have been extensively utilized to study microvasculature assembly [61]–[65], vSMC responses [66], [67] and multicellular organization [68], we chose to use fibrin as the matrix material to prepare hydrogel microfibers as a template for this study. Using the electrostretching approach, fibrin hydrogel microfibers were prepared and exhibited longitudinally aligned nanotopography (**Fig. 1A**). This is an important feature as sub-micron (<1 µm, but greater than 100 nm) scale topographic features have been shown to increase EC adhesion, migration, and orientation [4]–[7].

The seeding of ECFCs was facilitated by continuous rotation (**Fig. 1A**). After 24 hrs, ECFCs attached to the microfibers throughout the surface (**Fig. S1**). Within 5 days in culture, ECFCs were found to be elongated and aligned longitudinally with the microfibers as indicated by F-actin staining. ECFCs covered the microfiber surface continuously, and exhibited typical membrane expression of endothelial markers VEcad and CD31, and cytoplasmic expression of von Willebrand factor (vWF, **Fig. 1B–D**), demonstrating that fibrin microfibers support the adhesion and culture of ECFCs.

### ECM deposition from ECFCs on fibrin microfibers

**Figure 2:**
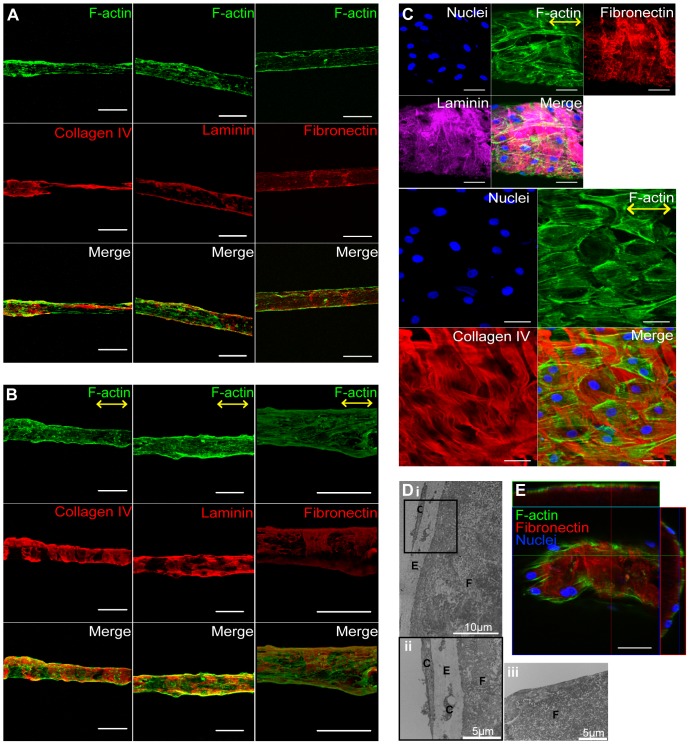
ECFCs deposit ECM circumferentially on fibrin hydrogel microfibers. Confocal z-stack image reconstructions of ECFCs on fibrin microfibers after (**A**) 1 and (**B**) 5 days in culture. Scale bars are 200 µm. (**C**) High magnification confocal images of laminin, fibronectin and collagen IV wrapping around the fibrin microfibers. (**D**) TEM images of cross-sectional slices of a cell-fibrin microfiber construct after 5 days in culture (i–ii) with cells and (iii) without cells. (ii) is a higher magnification image for the boxed area in (i). F  =  Fibrin; E  =  ECM; C  =  Cells. (**E**) Cross-sectional projections of confocal z-stack images of ECFCs on fibrin microfibers after 5 days. F-actin (phalloidin) is shown in green, ECM proteins (collagen IV, laminin, fibronectin) in red or magenta, and nuclei in blue. Yellow arrows indicate the direction of nanotopography on microfiber surface. A n≥2, B–E n≥5 per stain with quadruplicates.

While the importance of ECM deposition in vascular development has been recognized, few studies have looked at ECM production by the endothelium. Previously we demonstrated that ECFCs deposit collagen IV, fibronectin and laminin in an organized web-like structure when cultured on Petri dishes [58]. We therefore sought to characterize the ECM protein deposition by the endothelium on hydrogel microfibers. We found that ECFCs seeded on fibrin microfibers deposited laminin, collagen IV, and fibronectin after one day in culture (**Fig. 2A**). On Day 5, ECFCs completely covered the fibrin microfiber, and abundant ECM deposition was observed (**Fig. 2B**). In contrast to what we observed previously on Petri-dishes [58], the ECM proteins deposited by ECFCs on hydrogel microfibers were organized; laminin, collagen IV, and fibronectin wrapped around the microfibers, perpendicular to the EC orientation, along the microfiber's circumference (**Fig. 2C** **; Fig. S2; Video S1**). Moreover, these ECM structures appeared to be distributed either below or among the ECFCs (**Fig. 2D–E**, resembling the basal lamina found in native microvessels. It should be noted that similarly to what we observed on Petri-dishes [58], ECFCs did not express or deposit collagen I (data not shown).

### ECM deposition from ECFCs on fibrin sheets and PES fibers

**Figure 3:**
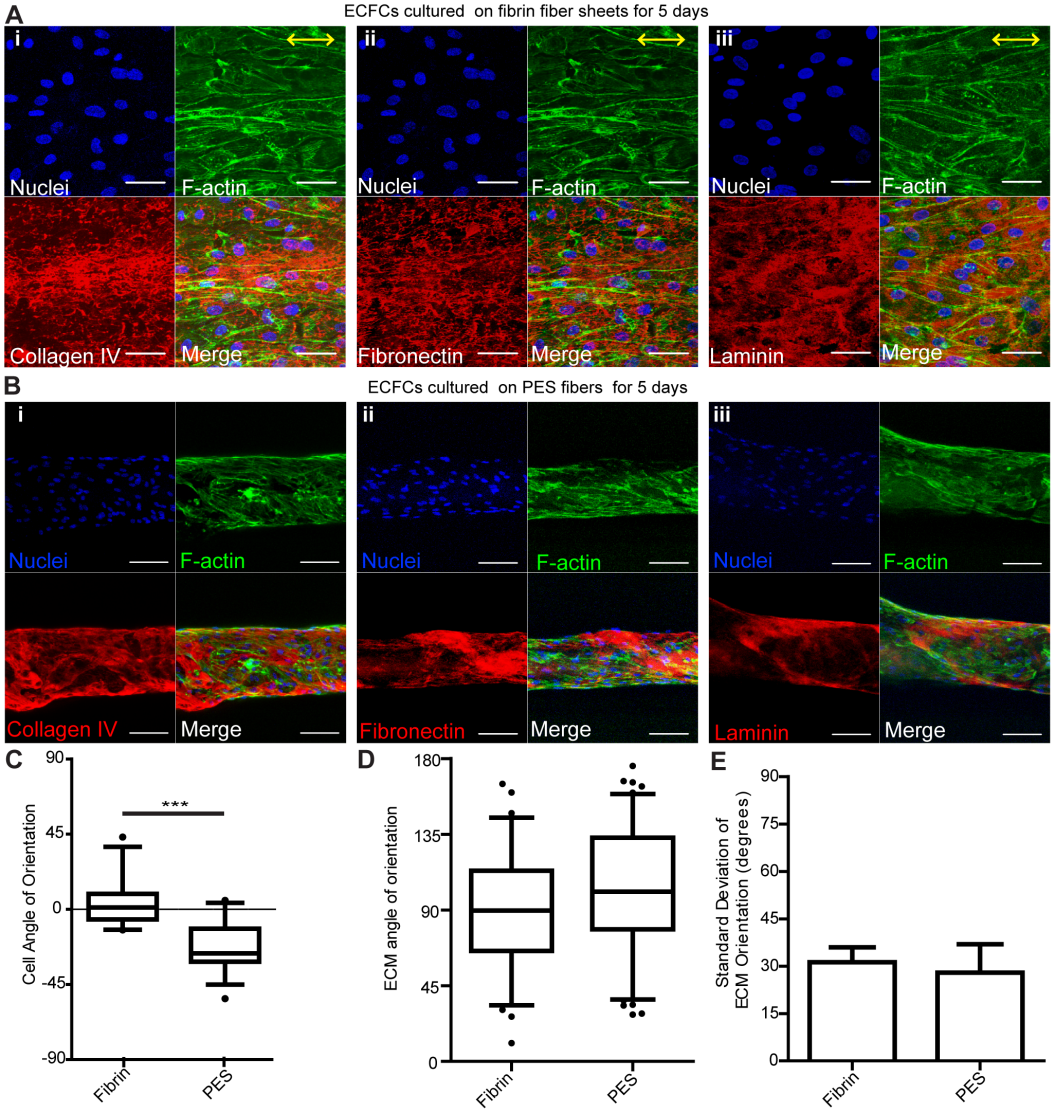
Nanotopography and geometry differently effect ECM organization. Confocal z-stack image reconstructions of (**A**) ECFCs on 2D fibrin sheets after 5 days in culture. Yellow arrows indicate the direction of nanotopography. Scale bars are 50 µm. n = 2 with duplicates (**B**) ECFCs on PES 3D fibrin-coated fibers with random non-aligned topography after 5 days in culture. Scale bars are 100 µm. n≥4 with quadruplicates. Actin filaments (phalloidin) are shown in green, ECM proteins (collagen IV, laminin, fibronectin) in red, and nuclei in blue. Box-and-whisker plots showing ECFC (**C**) and ECM (**D**) angle of orientation on PES and fibrin hydrogel microfibers after 5 days in culture. (**E**) Standard deviation of ECM angle of orientation. Error bars represent 5–95% confidence intervals. Significance levels in the mean represented by ***p<0.001. n≥2 with quadruplicates.

It was previously demonstrated that line-grating topography influences EC adhesion, alignment, and elongation [4]–[7]. To probe if the aligned nanotopography of the fibrin microfibers is responsible for ECFC alignment and coordinated deposition of laminin, collagen IV, and fibronectin, we first examined their deposition on flat (2D) fibrin sheets with a similar aligned nanotopography (**Fig. S3A**) as our hydrogel microfibers, but varying the dimensionality and cylindrical shape of our scaffold. As expected, ECFCs seeded on fibrin sheets were effectively aligned with the nanotopography. However, the collagen IV, fibronectin and laminin produced by ECFCs exhibited a random organization, as opposed to the perpendicular orientation with respect to cell alignment observed in the 3D microfibers (**Fig. 3A**). This result indicates that the microfiber geometry may be crucial to the specific organization of ECM molecules secreted by ECFCs.

We next utilized electrospun PES fibers coated with fibrin to generate fibers that maintain the dimensionality and geometry of our fibrin microfibers but with a random nanotopography (**Fig. S3Bi**). Note that before coating the PES fibers with fibrin, the surface of PES fibers is smooth (**Fig. S3Bii**), however, an uncoated PES fiber is not bioadhesive, and we could not detect any ECFC attachment after seeding (data not shown). Furthermore, it does no present the same bioactive substrate to the ECFCs as the fibrin fibers. Therefore, we coated the PES fibers with fibrin, resulting in the random, non-aligned nano-topography of coating presented in **Fig. S3Bi**. Such PES fibers have a similar diameter (240±45 µm; data not shown) as fibrin microfibers and thus enable us to investigate whether the uniaxial alignment topography contributes to the unique cellular activity and ECM organization. ECFCs were seeded according to the same protocol used for fibrin hydrogel fiber seeding. Similarly as observed in **Fig. 2B**, ECFCs completely covered the PES microfibers and deposited ECM molecules after 5 days of culture (**Fig. 3B**). While ECFCs grown on the PES fibers did not necessarily have a random orientation on the PES fibers, and in fact often exhibited a partial diagonal orientation (**Fig. 3C**), the ECFC-deposited ECM proteins were still found to wrap around the PES fiber in a similar manner to ECFC-deposited ECM on fibrin microfibers, as evidenced by measuring the angles between ECM ribbons and the fiber's longitudinal axis (**Fig. 3D**). Note that the average angle in both cases is close to 90°, demonstrating perpendicular alignment, and that the distribution (represented by the height of the boxes and also shown as standard deviation in **Fig. 3E**) is small, signifying most ECM ribbons had an orientation close to 90°.

### ECM organization: dependence on ECFC alignment and microtubule organization

**Figure 4:**
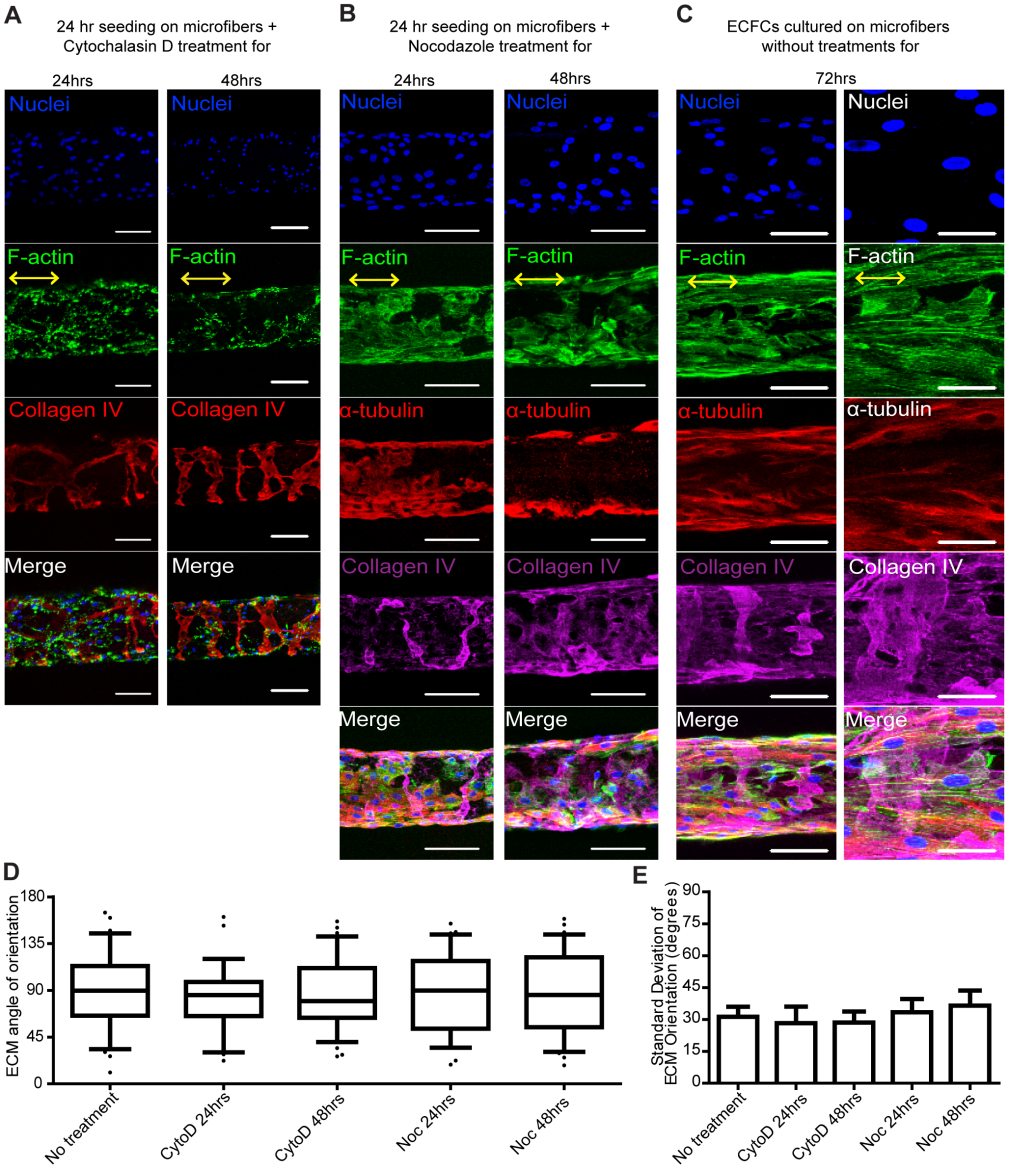
Disrupting actin and microtubule organization does not affect ECM organization. Confocal z-stack image reconstructions of ECFCs seeded on fibrin microfibers for 24 hrs followed by treatment with (**A**) cytochalasin D or (**B**) nocodazole for 24 hrs and 48 hrs in culture. (**C**) Low (left) and high (right) magnification of ECFCs seeded on fibrin microfibers for 72 hrs without drug treatment, serving as control. F-actin (phalloidin) is shown in green, microtubules (α-tubulin) in red, ECM proteins (collagen IV or fibronectin) in red or magenta, and nuclei in blue. Yellow arrows indicate the direction of nanotopography. Scale bars are 100 µm except of high magnification in C that is 50 µm. (**D**) Box-and-whisker plots and (**E**) standard deviation of ECM angle of orientation. Error bars represent 5–95% confidence intervals. n = 2 with quadruplicates.

We next sought to determine whether ECFC actin filament alignment and microtubule organization direct ECM organization. Towards this, ECFCs were seeded on fibrin microfibers and after 24 hrs an actin destabilization agent, cytochalasin D, was added to the culture media. We found that the unique wrapping arrangement of the deposited ECM molecules around the fibrin microfibers was still present after 24 and 48 hrs of treatment (**Fig. 4A** and **Fig. S4A**). No difference was found in the average angle of ECM orientation or in the variance of all angles measured (**Fig. 4D–E**). It should be noted that ECM deposition was observed also in control treatments of ECFCs in Petri dishes (**Fig. S4B**) and that similar organization of wrapping ECM was observed when cytochalasin D was applied through the entire 3 days of culture (**Fig. S4C**).

Likewise, when nocodazole, a microtubule polymerization disturbing agent, was added to the culture media at 24 hrs after ECFC seeding on microfibers, a similar ECM wrapping pattern was observed (**Fig. 4B** **; Fig. S5A**) even though microtubule formation was disrupted compared to samples with no drug treatment (**Fig. 4C**). Similarly, no difference was found in the average angle of ECM orientation or in the variance of all angles measured (**Fig. 4D–E**). In addition, ECM deposition was also observed in the 2D control group with the same treatment (**Fig. S5B**), and similar organization of wrapping ECM was observed when Nocodazole D was applied through the entire 3 days of culture (**Fig. S5C**).

Overall, while treatment of ECFCs seeded on the nanopatterned microfibers with either cytochalasin D or nocodazole effectively altered actin and microtubule organization, respectively, it did not alter the wrapping organization of the deposited ECM molecules as compared to control cultures (**Fig. 4**). Also, even after only 3 days of culture when the endothelium layer was not always confluent and therefore the cell density was lower, ECM organization was still found to be circumferential (**Fig. 4C–E**, **Fig. S6**), suggesting there is no effect of cell density on ECM organization.

### ECM organization: dependence on microfiber curvature

**Figure 5:**
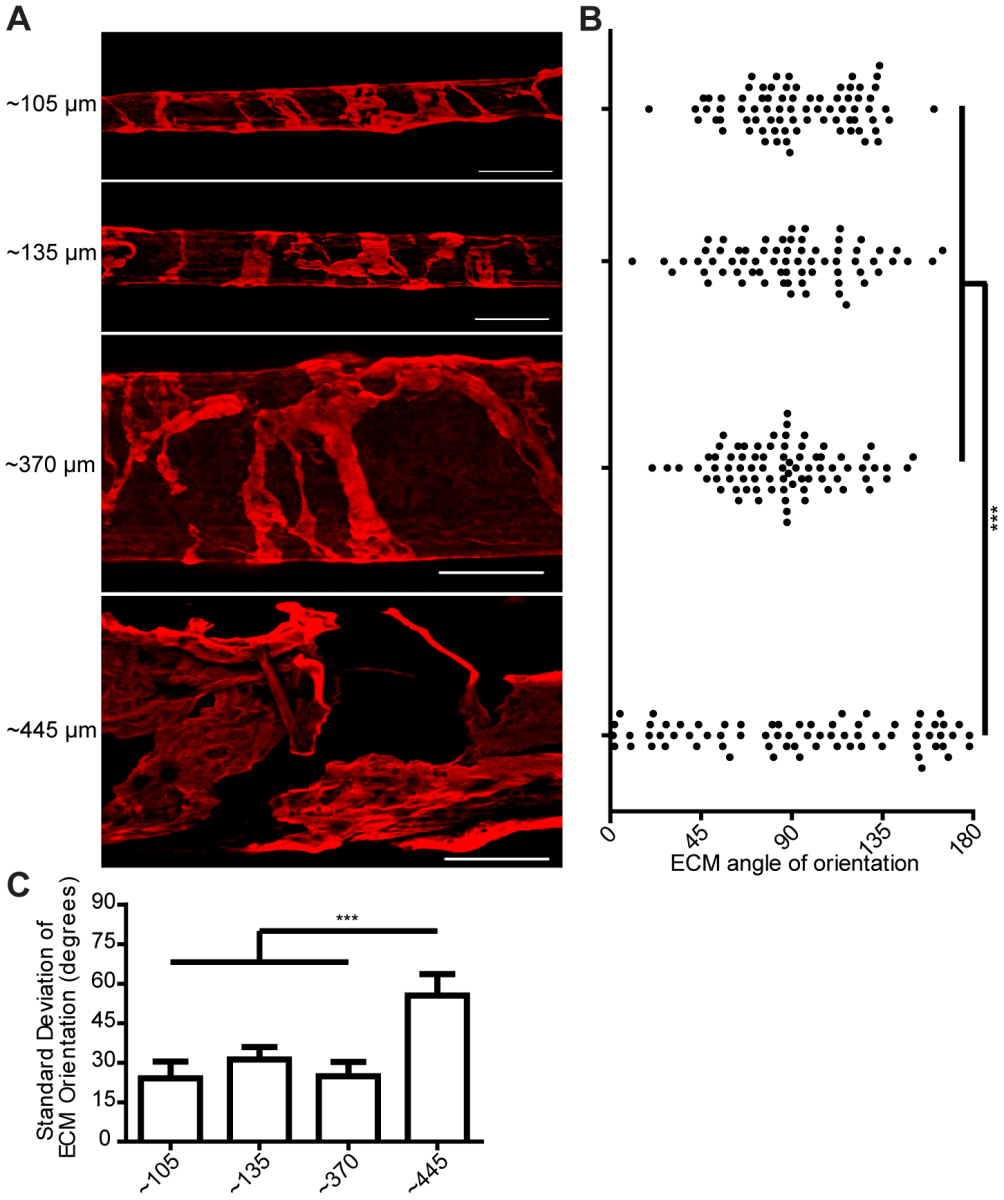
Microfiber curvature influences ECM organization. (**A**) Confocal z-stack image reconstructions of collagen IV deposition on fibrin microfibers with different diameters. Scale bars are 200 µm (**B**) Scatter plot and (**C**) standard deviation of ECM angle of orientation on microfibers with different diameters. Error bars represent 5–95% confidence intervals. Significance levels in the distribution represented by ***p<0.001. n = 2 with quadruplicates.

As our data indicated that the ECM organization is independent of the cytoskeleton organization of the ECFCs, but is influenced by the geometry of the microtubular structure, we next tested the hypothesis that the diameter of the tubular structure modulates the ECM organization. We prepared hydrogel microfibers of different sizes by varying the collection time of the electrostretching process, thus changing the number of nanofibers in each microfiber bundle. Fibrin microfibers with an average diameter of 107.1±11.7 µm, 136.1±12.1 µm, 372.0±27.3 µm, and 443.4±30.6 µm were prepared. These microfibers were processed similarly and thus exhibited similar nanotopographical alignment [59], inducing alignment of ECFCs with the longitudinal axis of the microfibers on all sizes (data not shown). However, we noticed that decreasing organization of the ECM molecules was apparent on microfiber's with large diameters (**Fig. 5A**). Measuring the angles between ECM ribbons and the microfiber's longitudinal axis revealed that microfibers with diameters smaller than ∼400 µm had average angles close to 90° with a small distribution, signifying perpendicular orientation. On the largest diameter tested (443.4±30.6 µm), we observed a non-circumferential ECM organization as evidenced by a significant increase in the distribution of the angles between ECM ribbons and the microfiber's longitudinal axis (**Fig. 5B**). Since values measured range from 0° to 180°, a perfectly random distribution of angles would average to the mean of 90° as well. However, a sample with ECM wrapping would have most of these values close to 90°, having a small variance compared to a sample presenting random ECM orientation. Indeed, the largest microfibers resulted in ECM angles with a markedly higher standard deviation compared to the smaller microfibers (**Fig. 5C**).

We also observed that when collagen IV has a wrapping organization, the individual collagen nano-fibrils also seem to follow this macroscopic circumferential alignment at the nano-scale (**Fig. 2C, S2B**). Further analysis revealed this was also true for microfibers of ∼105 to ∼370 µm, and that the largest microfibers tested (∼445 µm) did not have a distinguishable collagen nano-fibril orientation (**Fig. S7**).

### vSMCs attachment and new ECM deposition on ECFC-seeded microfibers

**Figure 6:**
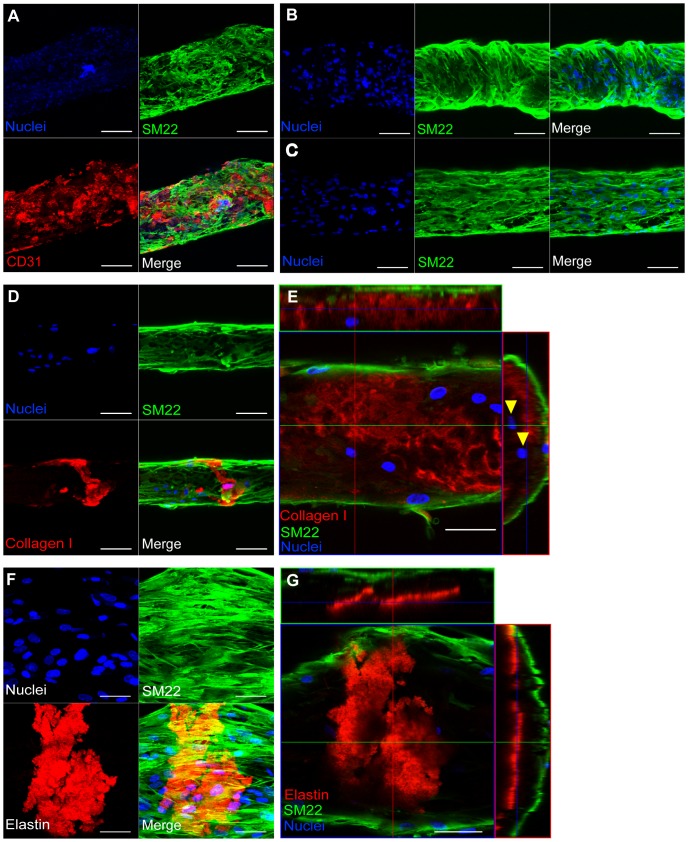
Co-cultured vSMCs deposit new ECM. Confocal z-stack image reconstructions of fibrin microfibers seeded with ECFCs followed by (**A**) co-culture of vSMCs for 2 days. n = 2 with quadruplicates. Scale bars are 200 µm. Co-cultured vSMCs for 3 days showing (**B**) wrapping and (**C**) aligned arrangement. Scale bars are 100 µm. (**D**) Collagen I deposited by co-cultured vSMCs after 3 days in co-culture. Scale bars are 100 µm. (**E**) Cross-sectional projection of confocal z-stack images of vSMCs after 5 days in co-culture. Arrowheads indicate SM22^−^ cells. Scale bars are 50 µm. B–E n≥3 with quadruplicates. (**F**) Confocal z-stack image reconstruction and (**G**) cross-sectional projection of co-cultured vSMCs after 5 days in co-culture. n = 2 with quadruplicates. Scale bars are 50 µm. SM22 is shown in green, CD31 in red, collagen I and elastin in red, and nuclei in blue.

One of the advantages of our fibrin microfiber system is the opportunity to co-culture vSMCs towards the study of their interactions with the endothelial layer as well as the deposition of ECM components that compose the tunica media. vSMCs seeded on ECFC-coated fibrin microfibers attached and grew on the ECFC layer, forming a bilayer cellular construct (**Fig. 6A**). Occasionally, vSMCs were found to have a random orientation on the structures (**Fig. 6A**), and in some instances they wrapped around the microfibers (**Fig. 6B**. However, more often they aligned with the longitudinal axis of the microfibers (**Fig. 6C**). More importantly, vSMCs cultured for 3 and 5 days deposited collagen type I and elastin (**Fig. 6D–G**), located beneath the vSMC layer and above SM22 negative cells, the ECFCs (**Fig. 6E, G**).

## Discussion

The cellular and ECM composition, as well as their specific structural organization, varies greatly in blood vessels of different type, size, and function. While postcapillary venules (10–30 µm) are formed by ECs and their basal lamina, along with scattered pericytes, and are semipermeable like capillaries [69], larger venules (larger than 50 µm in diameter) have a muscle layer and a thin adventitia [69]. Both arterioles of 100 µm to 300 µm in diameter and muscular arteries (300 µm to 1 cm) have an aligned endothelium sitting on its basal lamina, surrounded by a layer of connective tissue. This layer is followed by a well-defined fenestrated internal elastic membrane (with ECM occupying void spaces in the fenestrae) and a developed tunica media composed of several layers of circumferentially oriented vSMCs and ECM (mainly collagenous and elastic fibrils). These arteries are the main vessels in charge of restricting blood flow to capillary beds via vSMC constriction in response to neural or chemical stimuli [69], [70]. Even though the ECM is known to be circumferentially oriented in the media, the specific alignment of each layer in the tunica intima remains unclear; several studies report a mix of circumferential orientation close to the lumen and axial orientation close to the media [10]–[12], [15], [17]. However, it is known that circumferentially aligned ECM is necessary for vessels to be able to withstand the circumferential stress resulting from the distending pressure of blood flow. These facts highlight the importance of having both circumferential alignment of ECM and several layers of vSMCs, an aspect not found in current microvasculature models. Furthermore, the organization of other ECM components, such as fibronectin and laminin, remains widely uninvestigated.

Most current approaches for the *in vitro* study of the microvasculature in a 3D setting use hydrogels and scaffolds embedded with vascular cells, which are subject to spontaneous capillary bed formation [35]–[38], [60], [71]–[81], or use micropatterned hydrogels to generate organized microvasculature structures [46]–[48]. While these approaches are instrumental for studying angiogenic processes, they provide only partial control over the topographical cues presented to the cells by the ECM and possess limited opportunities to create and investigate multi-cellular vascular structures with proper ECM organization.

Our goal in this study was to create a model of the microvasculature that recapitulated both cellular and ECM organization, towards the understanding of microvasculature development and utilization of the model for regenerative medicine applications in the future. Towards this, we utilized electrostretched microfibers designed to generate a micro-cylindrical mold with a line-grating nanotopography to enable both endothelial layer organization and co-culture of supporting mural cells such as vSMCs. We utilize microfibers with diameters ranging from 100 to 450 µm, corresponding to a poorly studied range of vasculature in the body, namely venules and arterioles. Furthermore, in the existing models of microvasculature, the deposition and organization of ECM proteins by endothelial cells has not been studied. Moreover, the full investment of mural cells on the endothelium of microvascular models has been challenging, due in part to the use of endothelial-lined void spaces in most models [21], [22], which introduces a cell migration barrier for mural cell investment. In contrast, our model allows not only high resolution studies of both cell and ECM organization; it allows introduction of mural cells after endothelial layer formation and enables full investment of these mural cells, recreating the media layer of microvasculature.

We first demonstrate that the aligned microfiber bundles can be utilized as a cylindrical platform to control the organized adhesion of ECFCs. ECFCs cultured on fibrin microfibers with diameter of 100–450 µm attached throughout the microfibers, creating a continuous monolayer over the entire microfiber with a distinctive elongated and mature morphology. The fibrin microfibers thus offer an innovative approach in which ECFCs are seeded on the surface of an electrostretched microfiber, as opposed to in the body of a nanofiber mesh, as conventional electrospun scaffolds have been used [38], [39], [82], [83]. This unique approach allows detailed control of the cellular assembly of microvasculature, as the fibrin microfibers present a blueprint with a unique aligned nanotopography around which ECFCs can adhere to.

Examining the ECFC-deposited ECM organization on the fibrin microfiber, we found that after 5 days in culture ECFCs deposit collagen IV, fibronectin and laminin wrapping in discrete circumferentially aligned segments on the microfibers, perpendicular to their macroscopic cellular alignment and intracellular cytoskeletal organization. This characteristic of ECFCs of depositing abundant ECM [58] that is assembled circumferentially on a micro-cylindrical mold recognizes an active role of the endothelium in the construction of the extracellular components of the microvasculature. It is important to note that in longer period cultures of ECFCs on fibrin microfibers (i.e. >10days) we observed full coverage of the structures by ECM (data not shown). However, at this time point the initial circumferential organization of the ECM became impossible to analyze due to several layers of ECM being deposited on top of each other. For quantification purposes, we analyzed cultures of ECFC when ECM organization was evident, before full coverage was achieved.

As this is the first time that circumferential wrapping of ECM is reported by ECFCs, we proceeded to elucidate whether the ECFC alignment or the cylindrical structure and 3D aspect of the scaffold had a direct effect on ECM organization. Towards this, we designed two different systems that varied these parameters. The first scaffold used was a flat fibrin sheet with the same nanotopography as our original microfibers, varying therefore only the shape and geometry of the scaffold. ECFCs were found to align with the nanotopography of the fibrin sheets, but the ECM was deposited with no distinguishable organization. The second scaffold used was a polymer fiber coated with fibrin, which maintains the microfiber's spatial geometry, but has a random surface topography. Here, while ECFCs were not induced to align with the fiber's longitudinal axis, they still deposited ECM wrapping circumferentially around the polymer fiber, similarly to ECFCs seeded on fibrin microfibers. Together, these results suggest that the cylindrical shape of the fibers, and not the cellular organization induced by the scaffold's nanotopography, is necessary for ECM circumferential deposition.

The cytoskeleton is known to regulate endothelial alignment [4], [7] and drive angiogenic responses through ECM-interactions [27], [84]. Thus, to further verify whether the ECFC alignment on the microfibers through actin and tubulin configuration is instrumental for their circumferential ECM deposition, we examined the effect of cytoskeleton re-arrangement of ECFCs seeded on fibrin microfibers. We found that inhibition of neither actin filament nor microtubule polymerization affected ECM circumferential organization around the fibrin microfiber. These results signify that ECM organized deposition from ECFCs is independent of their cellular organization. Furthermore, shorter culture time periods (day 3) which did not always have a confluent endothelium still resulted in wrapping ECM, suggesting an independence of cell density on ECM organization.

Overall, even though we demonstrate that the aligned topography of the fibrin microfibers induces ECFC alignment, the findings that either ECFCs seeded on PES fibers or ECFCs seeded on fibrin microfibers with disrupted actin and microtubule organization still produce wrapping ECM suggest that ECM organization is regulated by 3D geometric sensing of curvature, rather than by the scaffold's nano-topography. Further studies are required to elucidate the underlying mechanism that modulates ECM expression and deposition by ECFCs and whether this is specific for progenitors or similar for mature ECs.

We next set to elucidate whether the circumferential wrapping of the ECM depended on the curvature of the microfibers. While curvature of nano-scale features of ECM has been suggested to regulate cellular responses [85], its effect on cellular responses at the micronscale and during microvascular formation and organization has not been investigated. As mentioned above, the electrostretching approach allowed us to generate microfibers with uniform and tunable diameters while preserving the aligned nanotopography. As our focus is on the microvasculature, microfibers with diameter ranging from 100–500 µm were examined. We found that microfibers of up to ∼400 µm in diameter guide the organized wrapping of deposited ECM, while large microfibers resulted in a more random ECM organization. This observation is the first to suggest an effect of curvature on ECM organization, and opens the door to further studies to determine the underlying mechanism. These results further highlight the novelty of our system, which enables us to study the role of curvature at the microscale in contrast to studies using larger diameter templates to study ECM deposition of perivascular cells [86].

Thus, this work presents the unprecedented role of endothelium in producing circumferentially aligned ECM, and the first-time discovery of the effect of micron-scale curvature on ECM organization as deposited by vascular cells. These studies exemplify the potential of the newly developed model to date in the study of processes of microvascular development, and highlight the importance of correct ECM organization in the microvasculature, a characteristic often not analyzed in current models.

We acknowledge that ECFCs will initially have an inverted polarity due to the presence of the fiber where the luminal surface would be and an absence of a tunica media on top. The first step towards correcting this inverted polarity was obtaining full investment of vSMCs on top of the endothelium, which has been achieved, demonstrating the versatility of our system in allowing sequential and controlled introduction of mural cells and their deposited ECM. For this, vSMCs were introduced into the model after 5 days of ECFC culture on the fibrin microfibers. First, vSMCs were found to attach to the ECFC-seeded microfibers with organizations that varied from the random, sporadic attachment typical of muscular venules to the circumferential wrapping observed in arterioles under physiological conditions [15]–[17], [19], [69], [70], but always in a multilayer configuration as opposed to the monolayer formed by the ECFCs. The different morphologies observed are most likely a result of lack of pulsatile flow, which has been shown to induce circumferential wrapping of SMCs in different studies [87], [88]. Indeed, the lack of internal pressure in our system is more conducive to the generation of muscular venules, which have much lower blood pressures compared to arterioles and contain randomly oriented vSMCs [69].

The next step in correcting the polarity of the ECFCs in our model is obtaining a defined lumen to create a hollow microvascular vessel. Still, another unique advantage of our fibrin microfiber scaffold is its biodegradability; fibrin can be easily degraded in a controlled manner using plasmin in conditions that do not affect cell viability [89]. Ongoing studies explore the degradation of the fibrin microfibers to generate a hollow microstructure with a defined lumen towards their use for applications *in vivo*. Thus, our system further offers future opportunities to fully correct the initial ECFC polarity and to study flow-induced vSMC organization post fibrin core degradation

Finally, vSMCs deposited collagen I and elastin on the ECFC-seeded microfibers, below the vSMC layer, and above the ECFCs. These results signify that co-cultured vSMCs deposited ECM components that organize the subendothelial connective tissue and internal elastic lamina in between the endothelium and the tunica media, which is in accordance to native microvascular organization (endothelium/basal lamina – subendothelial connective tissue – internal elastic lamina – tunica media) [15]–[17], [19], [69], [70]. Overall, we demonstrate investment of vSMCs with ECFCs in our system and distinctive vSMC-ECM deposition. It should be noted that we performed co-culture experiments in ECFC media, which was found to best support both ECFC and vSMC viability. However, this medium is not optimized for vSMC culture and may reduce their deposition of ECM. In fact, current studies in our lab focus on optimizing this phenomena and delineating the organization of vSMCs through their interaction with the endothelial layer.

## Conclusion

Collectively, we establish a novel *in vitro* model that recapitulates key aspects in the cellular and ECM organization of the microvasculature. Using this model we are able to guide organized microvascular structure formation, inducing endothelial cell alignment and elongation, and demonstrating circumferential deposition of ECM proteins by ECFCs. Our system enabled the discovery of the role of vessel diameter on ECM organization during human microvascular growth, a biophysical phenomenon not studied to date. Furthermore, we demonstrate the ability of our system to support a step-wise vascular formation process via introduction of perivascular cells at varying time points, a current challenge in microvascular tissue engineering. This approach can be used towards the mechanistic understanding of human microvasculature assembly and stabilization in health and disease.

## Supporting Information

Figure S1 **ECFC attachment on fibrin microfibers.** Confocal z-stack image reconstructions of ECFCs seeded on fibrin microfibers after one day in culture. Actin filaments (phalloidin staining) are shown in green, CD31 in red, and nuclei are counterstained in blue. Scale bars are 100 µm.
(TIF)

Figure S2 **ECM deposition by ECFCs on fibrin microfibers.** High magnification confocal z-stack image reconstructions of ECFCs-seeded fibrin microfibers after 5 days in culture showing (**A**) wrapping ribbon-like organization of Collagen IV, laminin and fibronectin (in red; Scale bars are 50 µm) and (**B**) horizontal orientation of ECFCs with circumferential organization of the deposited Collagen IV (red). Yellow arrow indicates the direction of nanotopography. Scale bars are 100 µm.
(TIF)

Figure S3 **Topography of fibrin microfibers sheet and PES microfibers.** (**A**) SEM of critical-point dried fibrin fiber sheets showing aligned topography on the surface. Scale bar is 2 µm (**B**) SEM of critical-point dried PES microfibers (**i**) coated with fibrin showing random topography and (**ii**) uncoated showing smooth topography. Scale bars are 10 µm.
(TIF)

Figure S4 **Cytochalasin D treatment of ECFC-seeded on fibrin microfibers.** Confocal z-stack image reconstructions of ECFCs seeded on (**A**) fibrin microfibers or (**B**) on Petri dishes for 24 hrs followed by treatment with cytochalasin D for 48 hrs in culture. (**C**) ECFCs seeded on fibrin microfibers and treated immediately with cytochalasin D for 72 hrs of culture. F-Actin filaments (phalloidin) are shown in green, collagen IV in red, fibronectin or laminin in magenta, and nuclei in blue. Yellow arrows indicate the direction of nanotopography on fibrin microfibers. Scale bars are 50 µm in A–B and 100 µm in C.
(TIF)

Figure S5 **Nocodazole treatment of ECFCs seeded on fibrin microfibers.** Confocal z-stack high-magnification image reconstructions of ECFCs seeded on (**A**) fibrin microfibers or (**B**) Petri-dishes for 24 h followed by treatment with nocodazole for 48 h of culture. (**C**) ECFCs seeded on fibrin microfibers and treated immediately with nocodazole for 72 hrs of culture. F-Actin filaments (phalloidin) in green, microtubules (α-tubulin) in red, Collagen IV in magenta, and nuclei in blue. Yellow arrows indicate the direction of nanotopography on fibrin microfibers. Scale bars are 50 µm in A (left), B, and C (right); 20 µm in A (right); and 100 µm in C (left).
(TIF)

Figure S6 **ECM deposition by ECFCs on fibrin microfibers after 3 days.** Confocal z-stack image reconstructions at different magnifications of ECFCs-seeded fibrin microfibers after 3 days in culture showing non-confluent ECFCs with circumferential organization of the deposited Collagen IV (red). Yellow arrow indicates the direction of nanotopography. Scale bars are (A) 200 (B) 100 (C) 50 µm.
(TIF)

Figure S7 **ECM deposition by ECFCs on fibrin microfibers of different sizes.** High magnification confocal z-stack image reconstructions of ECFCs-seeded fibrin microfibers with different diameter after 5 days in culture showing Collagen IV in red. Scale bars are 50 µm. Yellow arrow indicates the direction of nanotopography.
(TIF)

Video S1 3D z-stack image sequence of a microfiber with ECFCs cultured for 5 days exhibiting wrapping collagen IV.
(AVI)

Table S1 Reagents and Antibodies used in this study.
(DOCX)
