# Supplementary figures and images for: A Novel In Vitro Model for Microvasculature Reveals Regulation of Circumferential ECM Organization by Curvature
Source: PLoS One. 2013 Nov 21;8(11):e81061. doi: 10.1371/journal.pone.0081061 (PMC3836741; doi:10.1371/journal.pone.0081061)

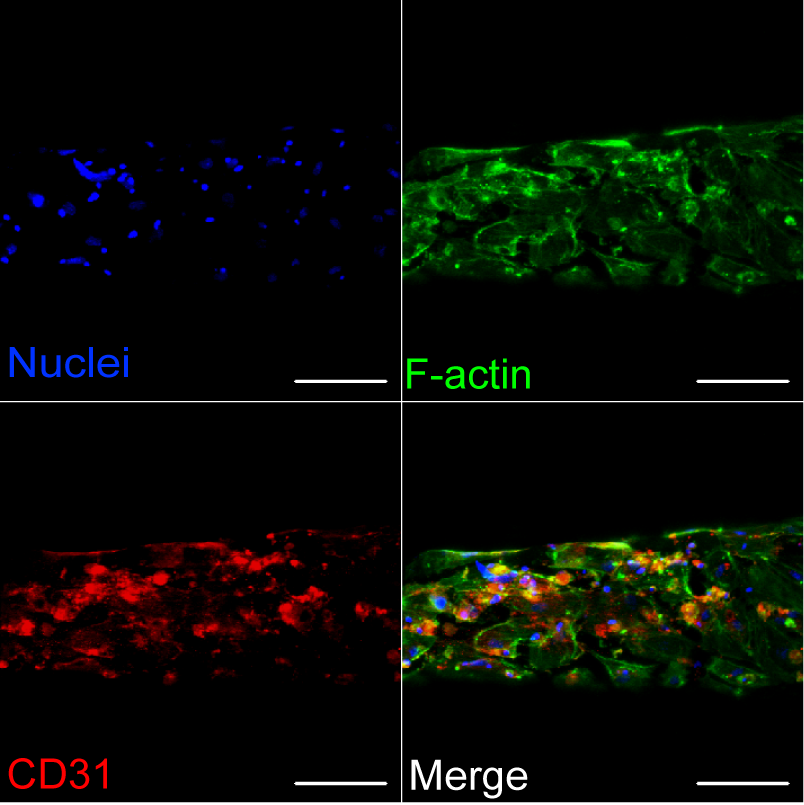

Supplement: Figure S1 — ECFC attachment on fibrin microfibers. Confocal z-stack image reconstructions of ECFCs seeded on fibrin microfibers after one day in culture. Actin filaments (phalloidin staining) are shown in green, CD31 in red, and nuclei are counterstained in blue. Scale bars are 100 µm. (TIF) [file pone.0081061.s001.tif]

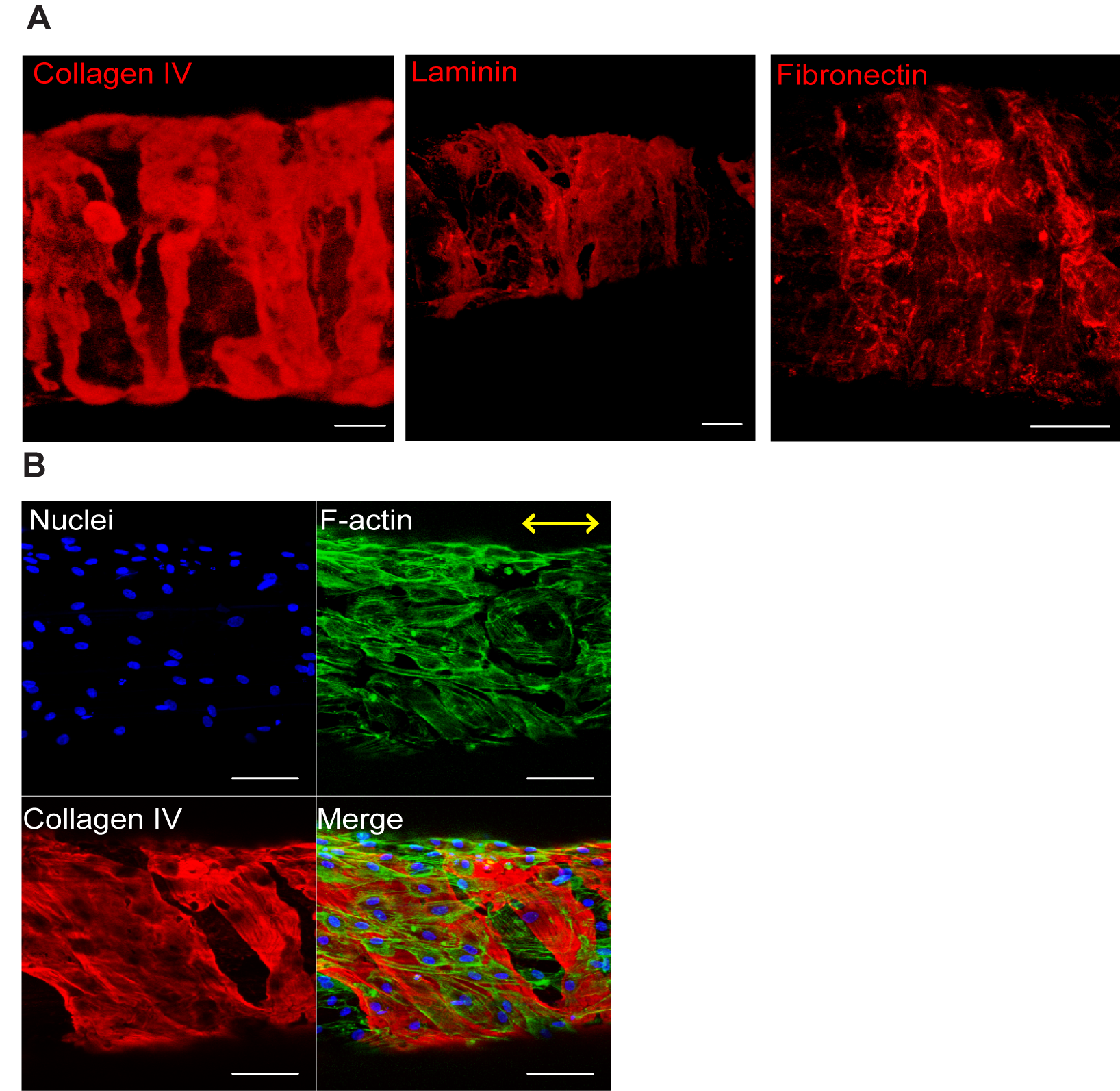

Supplement: Figure S2 — ECM deposition by ECFCs on fibrin microfibers. High magnification confocal z-stack image reconstructions of ECFCs-seeded fibrin microfibers after 5 days in culture showing (A) wrapping ribbon-like organization of Collagen IV, laminin and fibronectin (in red; Scale bars are 50 µm) and (B) horizontal orientation of ECFCs with circumferential organization of the deposited Collagen IV (red). Yellow arrow indicates the direction of nanotopography. Scale bars are 100 µm. (TIF) [file pone.0081061.s002.tif]

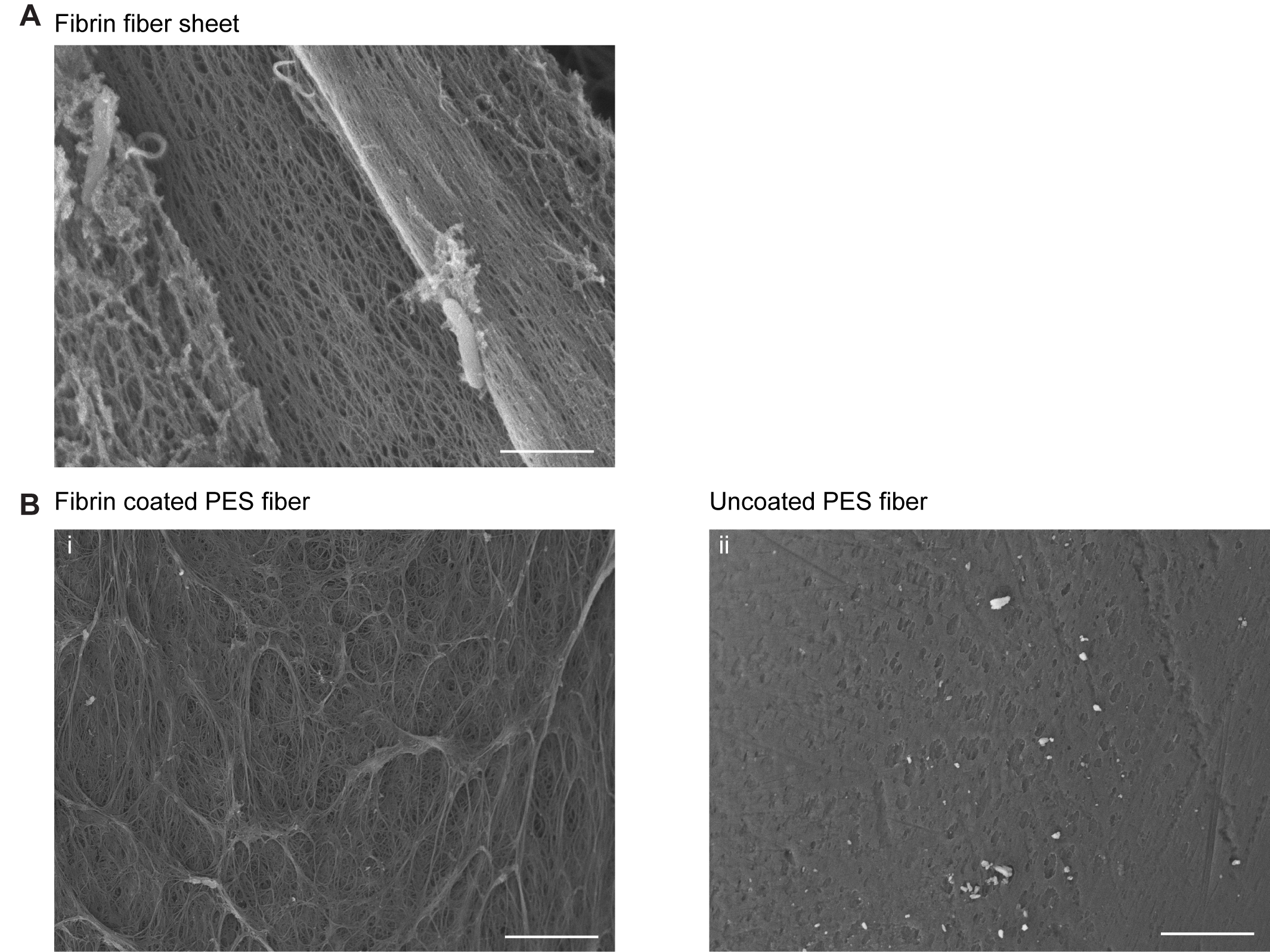

Supplement: Figure S3 — Topography of fibrin microfibers sheet and PES microfibers. (A) SEM of critical-point dried fibrin fiber sheets showing aligned topography on the surface. Scale bar is 2 µm (B) SEM of critical-point dried PES microfibers (i) coated with fibrin showing random topography and (ii) uncoated showing smooth topography. Scale bars are 10 µm. (TIF) [file pone.0081061.s003.tif]

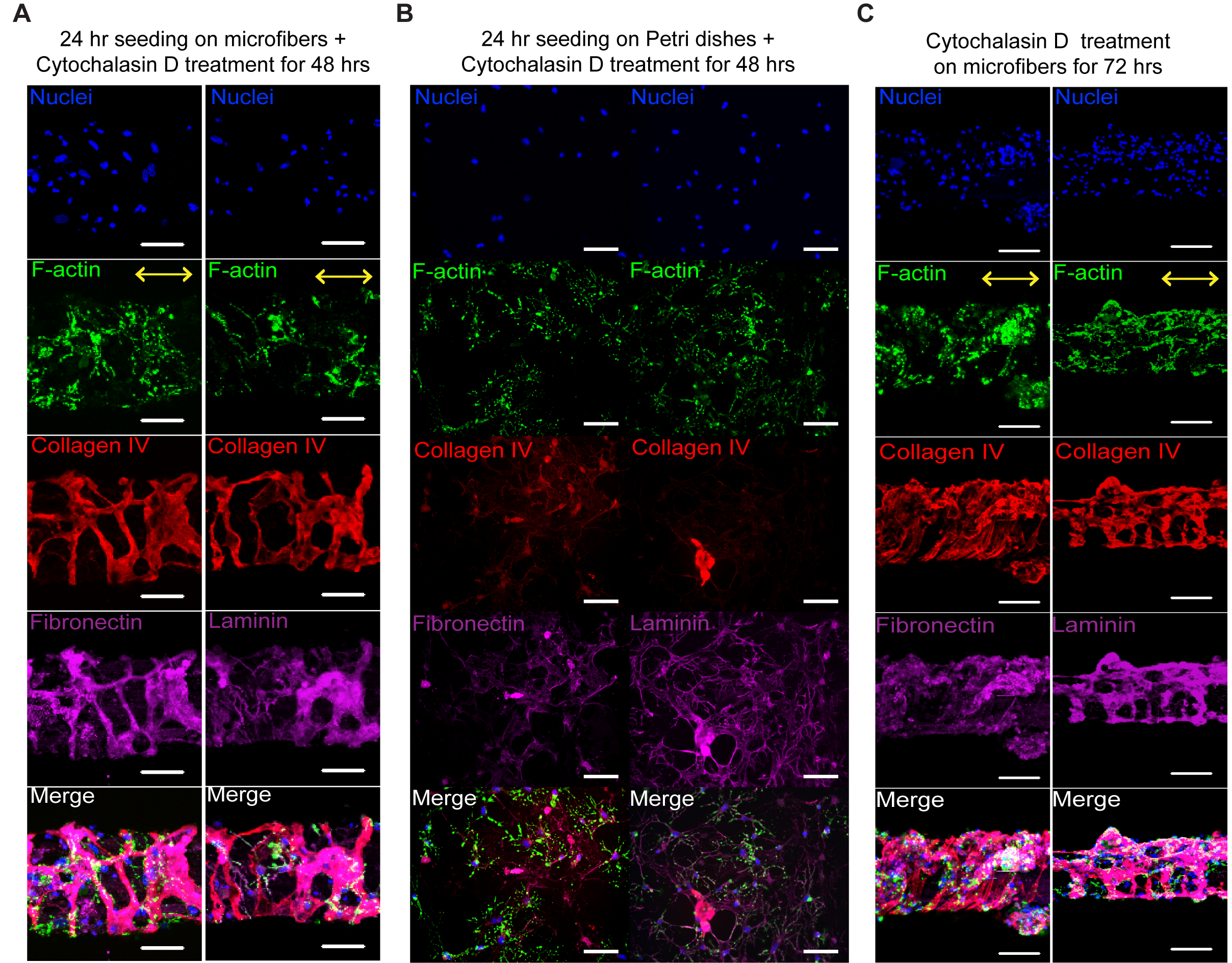

Supplement: Figure S4 — Cytochalasin D treatment of ECFC-seeded on fibrin microfibers. Confocal z-stack image reconstructions of ECFCs seeded on (A) fibrin microfibers or (B) on Petri dishes for 24 hrs followed by treatment with cytochalasin D for 48 hrs in culture. (C) ECFCs seeded on fibrin microfibers and treated immediately with cytochalasin D for 72 hrs of culture. F-Actin filaments (phalloidin) are shown in green, collagen IV in red, fibronectin or laminin in magenta, and nuclei in blue. Yellow arrows indicate the direction of nanotopography on fibrin microfibers. Scale bars are 50 µm in A–B and 100 µm in C. (TIF) [file pone.0081061.s004.tif]

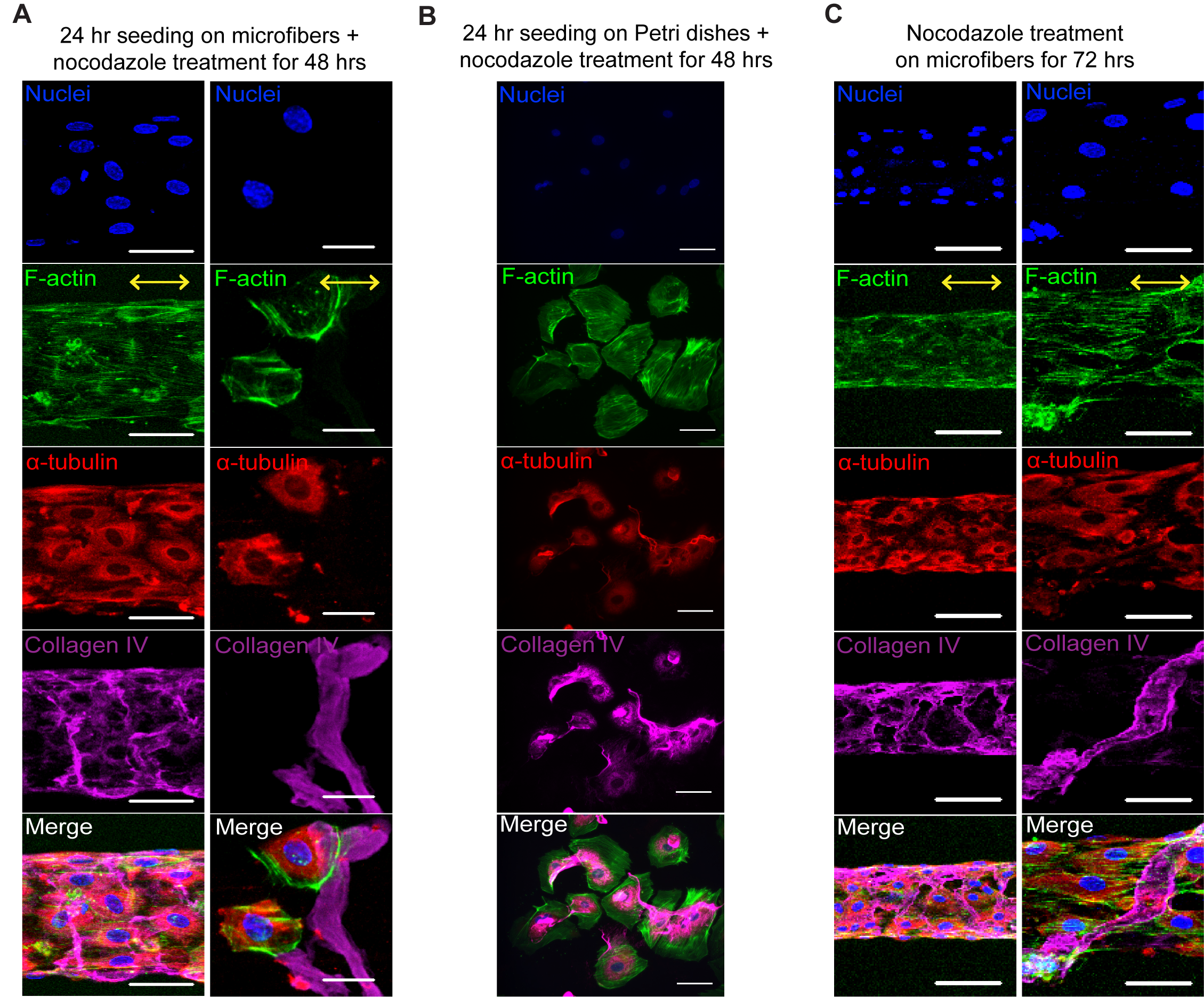

Supplement: Figure S5 — Nocodazole treatment of ECFCs seeded on fibrin microfibers. Confocal z-stack high-magnification image reconstructions of ECFCs seeded on (A) fibrin microfibers or (B) Petri-dishes for 24 h followed by treatment with nocodazole for 48 h of culture. (C) ECFCs seeded on fibrin microfibers and treated immediately with nocodazole for 72 hrs of culture. F-Actin filaments (phalloidin) in green, microtubules (α-tubulin) in red, Collagen IV in magenta, and nuclei in blue. Yellow arrows indicate the direction of nanotopography on fibrin microfibers. Scale bars are 50 µm in A (left), B, and C (right); 20 µm in A (right); and 100 µm in C (left). (TIF) [file pone.0081061.s005.tif]

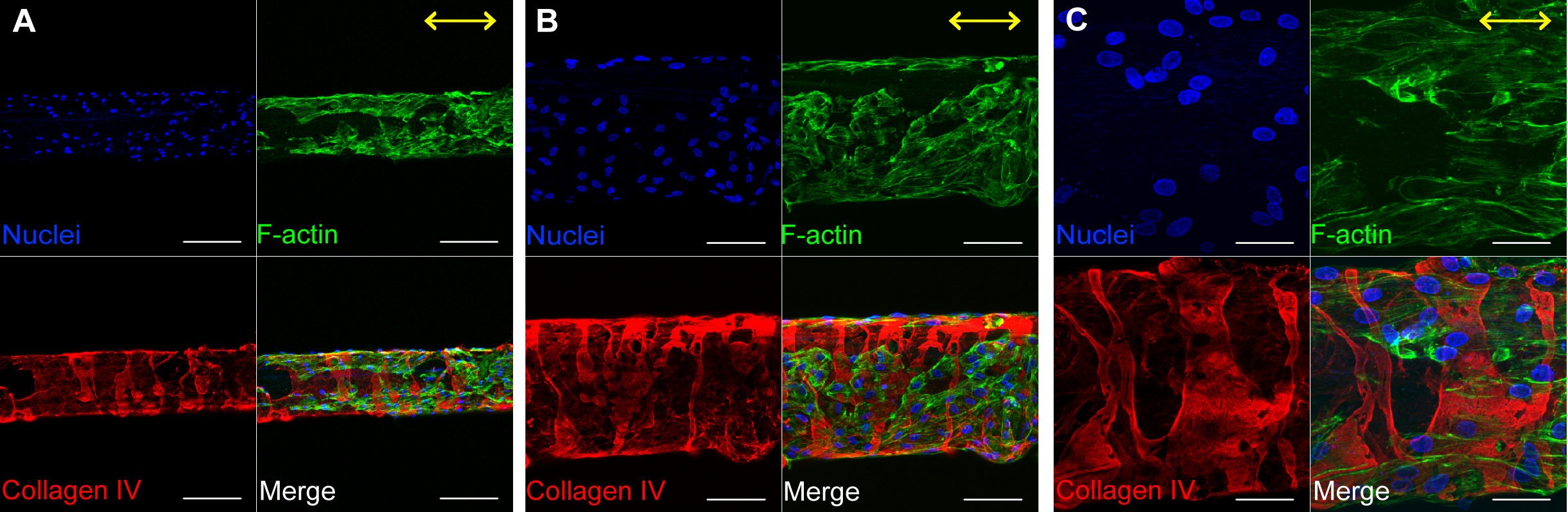

Supplement: Figure S6 — ECM deposition by ECFCs on fibrin microfibers after 3 days. Confocal z-stack image reconstructions at different magnifications of ECFCs-seeded fibrin microfibers after 3 days in culture showing non-confluent ECFCs with circumferential organization of the deposited Collagen IV (red). Yellow arrow indicates the direction of nanotopography. Scale bars are (A) 200 (B) 100 (C) 50 µm. (TIF) [file pone.0081061.s006.tif]

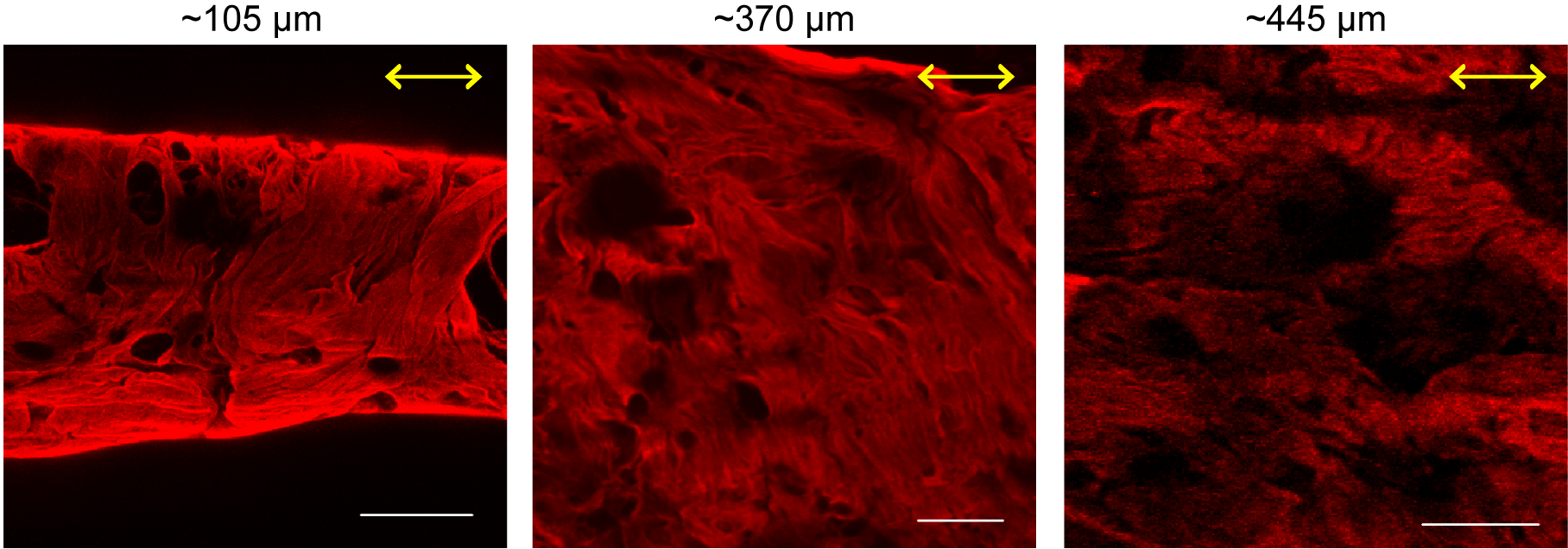

Supplement: Figure S7 — ECM deposition by ECFCs on fibrin microfibers of different sizes. High magnification confocal z-stack image reconstructions of ECFCs-seeded fibrin microfibers with different diameter after 5 days in culture showing Collagen IV in red. Scale bars are 50 µm. Yellow arrow indicates the direction of nanotopography. (TIF) [file pone.0081061.s007.tif]
