# Supplementary material for: A Novel In Vitro Model for Microvasculature Reveals Regulation of Circumferential ECM Organization by Curvature
Source: PLoS One. 2013 Nov 21;8(11):e81061. doi: 10.1371/journal.pone.0081061 (PMC3836741; doi:10.1371/journal.pone.0081061)
Supplement: Table S1 — Reagents and Antibodies used in this study. (DOCX) [file pone.0081061.s009.docx]

| **Reagent Type** | **Name** | **Host** | **Vendor** | **Dilution** | **Solvent** |
| --- | --- | --- | --- | --- | --- |
| *Microtubule disrupting agent* | Nocodazol | NA | Sigma-Aldrich | 3.3 μM | 0.1% DMSO |
| *Actin disrupting agent* | Cytochalasin D | NA | Sigma-Aldrich | 1 ug/mL | 0.1% DMSO |
| *Primary antibody* | CD31 | mouse | Dako | 1:100 | Dako antibody diluent |
|  | vWF | mouse | Dako | 1:100 | Dako antibody diluent |
|  | VEcad | mouse | Santa Cruz Biotechnology | 1:100 | Dako antibody diluent |
|  | SM22 | rabbit | Abcam | 1:200 | Dako antibody diluent |
|  | Collagen 1 | mouse | Abcam | 1:500 | Dako antibody diluent |
|  | Collagen 4 | mouse | Santa Cruz Biotechnology | 1:100 | Dako antibody diluent |
|  | Collagen 4 | rabbit | Santa Cruz Biotechnology | 1:100 | Dako antibody diluent |
|  | Elastin | mouse | Abcam | 1:100 | Dako antibody diluent |
|  | Fibronectin | rabbit | Sigma-Aldrich | 1:100 | Dako antibody diluent |
|  | Laminin | rabbit | Abcam | 1:100 | Dako antibody diluent |
|  | α-tubulin | mouse | Abcam | 1:500 | Dako antibody diluent |
| *Conjugated Antibody* | DAPI | NA | Roche Diagnostics | 1:1000 | PBS |
|  | Alexa Fluor 488 Phalloidin | shroom | Invitrogen | 1:50 | Dako antibody diluent |
| *Secondary antibody* | Alexa Fluor 488 anti- rabbit IgG | goat | Invitrogen | 1:1000 | Dako antibody diluent |
|  | Alexa Fluor 546 anti-mouse IgG | donkey | Invitrogen | 1:1000 | Dako antibody diluent |
|  | Alexa Fluor 546 anti-rabbit IgG | donkey | Invitrogen | 1:1000 | Dako antibody diluent |
|  | Alexa Fluor 647 anti-rabbit IgG | donkey | Invitrogen | 1:1000 | Dako antibody diluent |
